# Morphological control enables nanometer-scale dissection of cell-cell signaling complexes

**DOI:** 10.1038/s41467-022-35409-9

**Published:** 2022-12-20

**Authors:** Liam P. Dow, Guido Gaietta, Yair Kaufman, Mark F. Swift, Moara Lemos, Kerry Lane, Matthew Hopcroft, Armel Bezault, Cécile Sauvanet, Niels Volkmann, Beth L. Pruitt, Dorit Hanein

**Affiliations:** 1 Mechanical Engineering and Biomolecular Science and Engineering, University of California, Santa Barbara, CA USA; 2 Scintillon Institute, San Diego, CA USA; 3 Institut Pasteur, CNRS UMR3528, Structural Studies of Macromolecular Machines in Cellulo Unit, F-75015 Paris, France; 4 Institut Pasteur, Université de Paris, CNRS UMR3528, Structural Image Analysis Unit, Paris, France; 5 Present Address: Department of Chemistry and Biochemistry, and of Biomedical Engineering, University of California, Santa Barbara, CA USA

**Keywords:** Cryoelectron tomography, Developmental biology, Atomic force microscopy, Molecular biophysics, Cell signalling

## Abstract

Protein micropatterning enables robust control of cell positioning on electron-microscopy substrates for cryogenic electron tomography (cryo-ET). However, the combination of regulated cell boundaries and the underlying electron-microscopy substrate (EM-grids) provides a poorly understood microenvironment for cell biology. Because substrate stiffness and morphology affect cellular behavior, we devised protocols to characterize the nanometer-scale details of the protein micropatterns on EM-grids by combining cryo-ET, atomic force microscopy, and scanning electron microscopy. Measuring force displacement characteristics of holey carbon EM-grids, we found that their effective spring constant is similar to physiological values expected from skin tissues. Despite their apparent smoothness at light-microscopy resolution, spatial boundaries of the protein micropatterns are irregular at nanometer scale. Our protein micropatterning workflow provides the means to steer both positioning and morphology of cell doublets to determine nanometer details of punctate adherens junctions. Our workflow serves as the foundation for studying the fundamental structural changes governing cell-cell signaling.

## Abstract (web summary)

Micropatterning on electron microscopy grids enables control of cell positioning and arrangement. Here, the authors describe detailed nanoscale characterization of a micropatterning workflow for reproducible molecular characterization through cryo-ET

## Introduction

Functional cell–cell contacts are critical to both developing tissues as well as maintaining robust barrier function in healthy adult tissue. This connectivity is mediated by distinct epithelial cell–cell assemblies including tight junctions, gap junctions, and mechanical linkers (i.e., desmosomes and adherens junctions)^1,2^. The dynamic nature of developing tissues and the diverse external loads on epithelial barriers (e.g., stretching in the lung or gut) alter forces at cell–cell contacts that these junctions must respond and adapt to. Numerous studies have demonstrated how mechanical force transfer at these junctions regulate downstream epithelial function^2–12^. For example, tension on the E-cadherin protein of the adherens junction has been shown to influence mitotic polarity and facilitate migration^13–15^. To learn how epithelial junctions function and adapt to their environments, there is a need to visualize these complex structures in native cell–cell configurations at defined functional states. This challenging task requires nanometer resolution imaging of these dynamic structures hidden deep in cell–cell contacts at different positions along a cell–cell interface.

In the last few years, cryo-ET has become the state-of-the-art technique for visualizing protein complexes in the context of the native whole cell at high resolution. Unlike traditional transmission electron microscopy (TEM) staining techniques, cryo-ET enables imaging of cellular structures in a native cellular environment, maintaining their three-dimensional nanoarchitecture while fully hydrated^16,17^. In cryo-ET the sample is vitrified without staining, a tilt series of images from a multitude of angles is taken, and a three-dimensional (3D) reconstruction of the sample is calculated from the tilt series at nanometer resolution. Combining light microscopy with high resolution electron microscopy, for example, cryogenic correlative light and electron microscopy (cryo-CLEM) can be used to localize macromolecules and regions of interest by matching and aligning the 3D tomograms with fluorescent signals of light-microscopy images^18,19^. These revolutionary techniques have already provided insight into key cellular structures including desmosomes^20^ and cytoskeletal regulation^21–23^.

Despite the power of cryo-ET imaging, there are several limitations to this technique. The electron beam can only permeate thicknesses up to 300 nm, meaning thicker cellular structures cannot be imaged without additional sample processing. This is particularly problematic considering mammalian cells are often several microns in thickness. Even in thinner regions of epithelial cell–cell contacts, junctions are buried at unknown thicknesses. Cryogenic focused ion beam (cryo-FIB) milling can be used to remove regions of excess thickness and isolate thinner regions of a thick cellular structure (lamellae), enabling imaging of the cellular interior^24–29^, however precise spatial localization along the z axis of the region between the two modalities is not yet available. The other main limitation of Cryo-ET is that during tilt, many regions of the electron microscopy (EM) amenable substrate (the EM-grid) are obstructed from the field of view and thus cannot be imaged. Therefore, viable images can only be obtained from specific regions of EM-grids, resulting in low throughput of sample processing.

Protein micropatterning has been introduced as a method to spatially position cells on EM-grids and improve sample throughput for cryo-ET^23,30,31^. Cells can be positioned precisely in regions where there is no visual obstruction (i.e., center of grid and of mesh squares). Micropatterning combined with bio-passivation techniques can also reduce the adherence of cells or debris in regions of the grid that may obstruct viewing. These advantages allow a significant increase in the throughput of whole-cell cryo-ET. Furthermore, protein micropatterning provides unique opportunities for probing structure/function relationships by altering cellular shape and tension. Light microscopy, including fluorescence microscopy, has been instrumental in these findings. For example, controlling for cell geometry can influence stem cell fate and enhance maturity of cardiomyocytes^32,33^. Light microscopy can additionally be used to assess attachment, growth, and behavior of cells experiencing different mechanical inputs. Controlling for cell geometry via protein patterns offers the potential for whole-cell structure and function assays of cell–cell contacts not previously attainable^4,34^.

While we and others have published a few studies on the ability to pattern EM-grid substrates for electron tomography^30,31,35^ as well as attempting to increase the likelihood to obtain cell–cell contacts between protrusions^36^, the detailed characterization of these geometrically regulated substrates for functional assays remains unaddressed. Here, we have (i) adapted and optimized existing photomolecular adsorption techniques^37^ to micropattern EM grids in a completely contactless manner, (ii) provided molecular details and mechanical properties of the corresponding cellular microenvironment using a variety of high resolution imaging techniques, (iii) utilized these protein patterns to regulate the positioning and size of mature epithelial cell–cell contacts for high-throughput CLEM and cryo-ET imaging, and (iv) implemented cryo-FIB to isolate and segment geometrically regulated epithelial cell–cell contacts. We successfully achieved our goal of visualizing the nanometer details of adherens junctional puncta within mature cell–cell contact sites through harnessing contactless micropatterning, light microscopy, cryo-FIB, and cryo-ET. Our additional extensive characterizations of the mechanical properties of the hydrated carbon film and nanometer-scale details of the micropatterns beneath the cells offer new insight into functional regulation of micropatterned cells for cryo-ET studies. The results from this study offer enormous potential for elucidating the complex structure and function relationships of cell–cell junctions in the dynamic cellular environment.

## Results

### Contactless grid handling and micropatterning

**Fig. 1:**
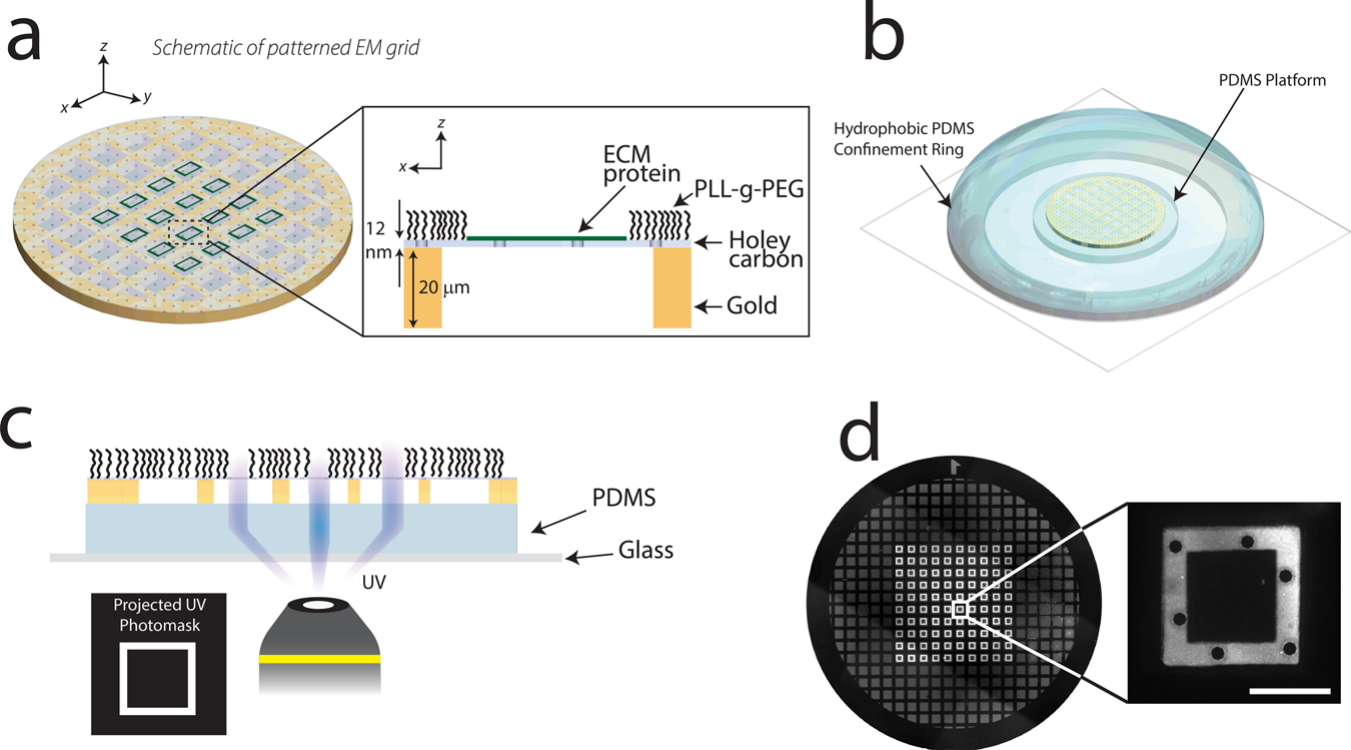
Micropatterning of holey carbon EM grids using photomolecular adsorption. **a** Schematic of patterned holey carbon film of gold mesh grid, with custom ECM patterns surrounded by a bio-passivation layer. **b** Grids are placed on a thin PDMS platform to eliminate physical contact with the carbon film, which is encompassed by a thicker PDMS ring to confine liquid on the grid. **c** Digital masks with custom patterns are projected through the carbon film to degrade the bio-passivation layer. **d** Adsorption of Oregon green gelatin to degraded regions of 50 × 50 µm squares within a 10 × 10 array. Scale bar is 30 µm.

Micropatterning of proteins on holey carbon electron microscopy grids is particularly difficult due to the fragility of the thin carbon film (~12 nm) that is deposited on a metal mesh. Our previous work overcame this challenge by utilizing contactless photomolecular adsorption to pattern ECM proteins^30^. In that work however, a carefully positioned thin silicone mask was used to adhere the grid to a substrate, which can make the carbon film vulnerable to rips or tears. To improve on previous work, we have now developed a contactless method for grid handling as well as micropatterning (Fig. 1). For patterning of ECM protein arrays on a conventional EM grid film (Fig. 1a), we leveraged custom cut PDMS shapes. A small circular PDMS platform adhered the gold metal mesh during plasma treatment, while a thicker outer PDMS ring created a hydrophobic barrier for a fully contained well of poly-l-lysine-grafted-polyethylene glycol (PLL-g-PEG) solution (Fig. 1b). This well served three purposes: (i) containment for subsequent rinse steps and protein incubation, (ii) minimal working volume and reagents, and (iii) sealing the buffer inside the well using a glass coverslip, allowing for robust shipping and handling procedures if necessary (Supplementary Fig. 1). We adapted previous techniques^30^ by exposing the UV digital mask directly through the thin PDMS base and exposing our custom pattern geometries on the holey carbon film (Fig. 1c). These geometries were patterned in a high-throughput manner as 10 × 10 arrays of micropatterns (Fig. 1d) and optimized to avoid stitching errors (Supplementary Fig. 2).

### Characterization of protein micropatterns

**Fig. 2:**
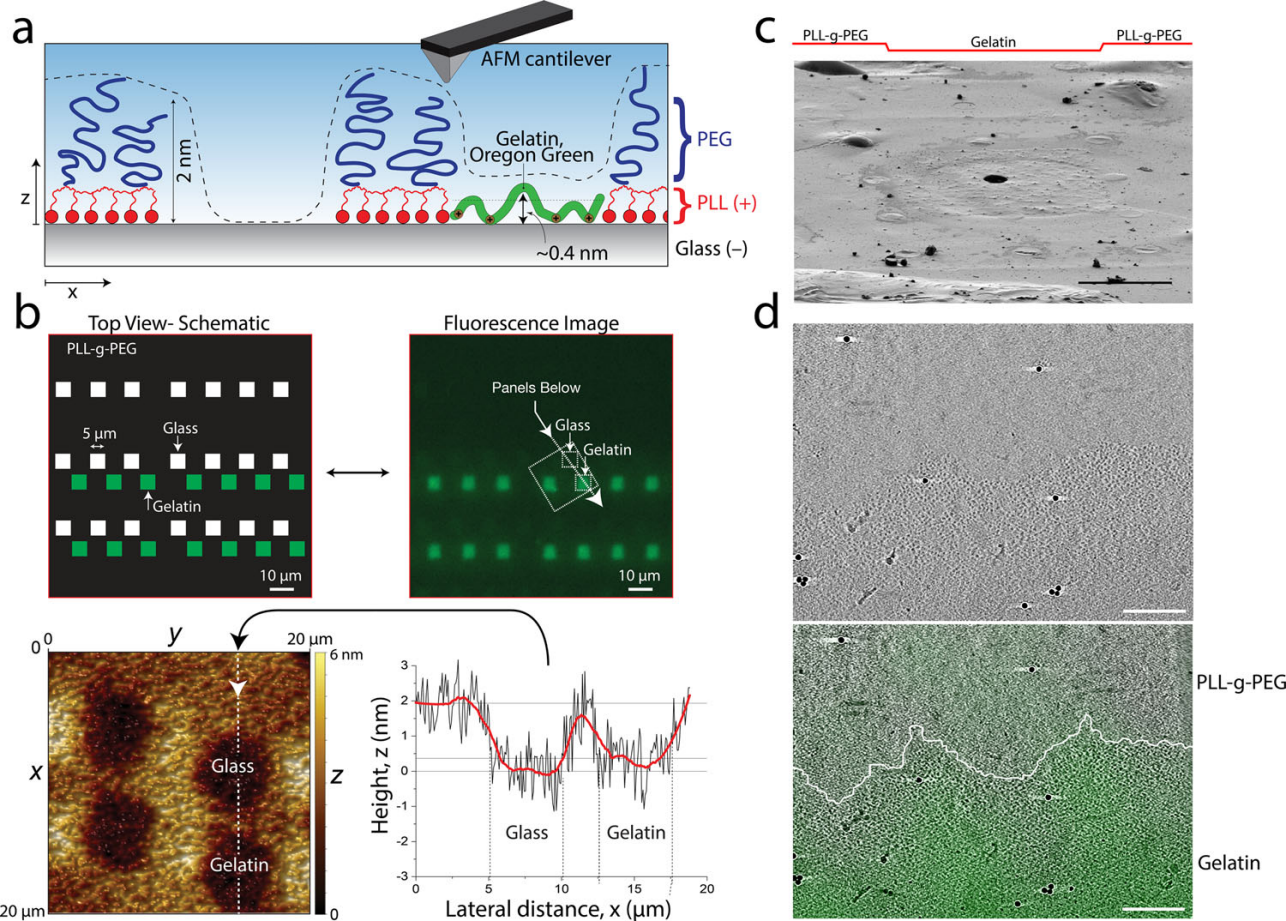
Nanoscale characterization of the micropatterning of holey carbon EM grids using photomolecular adsorption. **a** Schematic of an AFM cantilever scanning the surface of adsorbed gelatin surrounded by PLL-g-PEG. **b** A 2-step digital mask photopatterning method was used to create 5 µm squares with and without protein (upper panels). AFM probe scans clearly show squares with and without protein, which could be quantified with 0.5 nm resolution (lower panels). Representative images from triplicate measurements. **c** Custom ECM (Oregon green gelatin) 45 µm × 45 µm square pattern surrounded by PLL-g-PEG on the holey carbon film of a gold mesh grid imaged by the scanning electron microscope beam (at 2 kV) of the dual beam cryo-FIB instrument in cryo-SEM imaging mode. Representative image out of three repeats. The pattern is clearly visible as a depression, consistent with the atomic force microscopy results shown in **b**. **d** 2 nm thick slice of a representative tomogram of a patterned region. The fluorescence image of the ECM coating applied to the pattern was precisely overlaid on the cryo-ET image and the border between pattern and bio-passivated area was manually traced (white line). Scale bars 20 µm (**c**), 200 nm (**d**).

To determine whether our PLL-g-PEG bio-passivated layer was within the thickness range for electron-beam permeability, we utilized contact mode atomic force microscopy (AFM) on backfilled protein patterns on glass using similar photomolecular adsorption techniques (Fig. 2a). We exposed a two-layer digital mask of 5 µm solid square patterns on the glass slide, with an incubation of Oregon green gelatin between UV exposures (Fig. 2b). Therefore, the 2^nd^ exposure ablated identical 5 µm squares in the bio-passivated region with no subsequent protein backfill. These patterns were discernable using fluorescence imaging (Fig. 2c), which guided the placement of the AFM probe for surface scanning measurements. Our results indicated that the PLL-g-PEG bio-passivation layer was approximately 2 nm thick, while the gelatin ECM layer had a thickness of approximately 0.5 nm (Fig. 2d, e). These thickness measurements were approximately two orders of magnitude less than the maximum thickness constraint for our sample (<300 nm), indicating that neither the thickness of the ECM protein nor the bio-passivation layer would burden the sample thickness during electron beam transmission. Interestingly, the protein patterns differ enough in thickness and density from the surrounding PLL-g-PEG that they can be visualized by electron beam transmission (Supplementary Fig. 3). We further verified that these thickness measurements translated onto the ECM patterns of the holey carbon grid by performing cryo-SEM (Fig. 2f). In addition to pattern topography, we further characterized the lateral resolution of the patterns on the carbon film, which has not been assessed before, using cryo-CLEM (Fig. 2f). Reconstructed tomograms of the protein patterns demonstrated a clear boundary between the protein layer and the bio-passivation layer that is rather irregular at the nanometer scale despite the smooth appearance at light-microscopy resolution (Fig. 2g).

### Mechanical characterization of carbon film

**Fig. 3:**
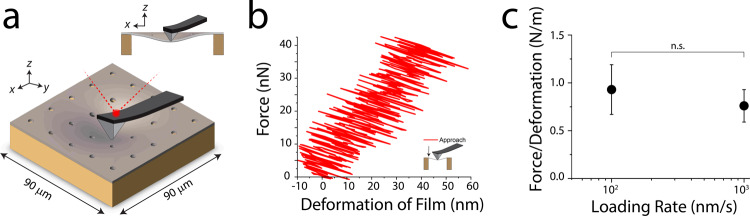
Mechanical characterization of carbon film cell substrate. **a** Schematic of carbon square of EM grid being loaded in the center by the AFM probe. **b** Example of a single distance/force curve from loading one of the grid squares at a loading rate of 100 nm/s (red-approach). **c** The slopes of force/deformation curves (determined by a total least square regression) were used to calculate the spring constant associated with the carbon film across 100 nm/s (*n* = 6) and 1000 nm/s (n = 6). Each n represents a measurement from a separate grid square, Our results show no significant change between these loading rates. Error bars represent s.d., the centers mark the mean values. Statistical significance between the data sets was determined using a two-tailed Student’s *t* test assuming equal variance.

The mechanical properties of cell substrates have been shown to influence cellular properties, including the structure and molecular tension at cell–cell contacts^38,39^. However, the rigidity of the hydrated carbon film on the electron microscopy grids used for cell culture is unknown. Therefore, we mechanically characterized the EM-grid squares to learn their rigidity in comparison to commonly used mechanobiology substrates (e.g., hydrogels, PDMS). We used an AFM in contact mode (see details under Carbon Film Force Deflection in Methods) to apply a force to the center of the carbon film over a grid square, i.e., to push the cantilever tip against the film (Fig. 3a). We performed this measurement for loading rates of 100 nm/s and 1000 nm/s to verify elastic behavior of the film. By modeling both the cantilever and carbon film as elastic springs in series under load, we calculated the spring constant of the deformed carbon film (Fig. 3b and Supplementary Fig. 4). The effective spring constant, *k*_*f*,_ for a grid square was found to be 0.85 ± 0.23 (N/m), independent of loading rate (Fig. 3c). We used this experimentally determined spring constant, *k*_*f*_, to approximate what equivalent material properties of a hydrogel would yield the same effective spring constant. For a gel having homogenous elastic modulus *E*_*gel*_ and Poisson ratio *ν*, the relationship of rigidity to stiffness can be modeled as an elastic half-space under vertical point load application (Eq. 1).

1 $$k=\frac{\pi \alpha {E}_{{gel}}}{\left(1-v\right)(1+v)}$$

Assuming a gel Poisson’s ratio of 0.48^40,41^ and a radius of adhesion (α) of 1 µm^42^, we estimate the spring constant of a grid square is equivalent to that of a gel having an elastic modulus *E*_*gel*_ of 207 ± 56 kPa. This value is comparable to polyacrylamide gels used for cell culture^41^. We attribute variability of this value to ambient noise in the measurements (Supplementary Fig. 5), as well as heterogeneity between squares of the EM-grid.

### Confinement of epithelial cell pairs

**Fig. 4:**
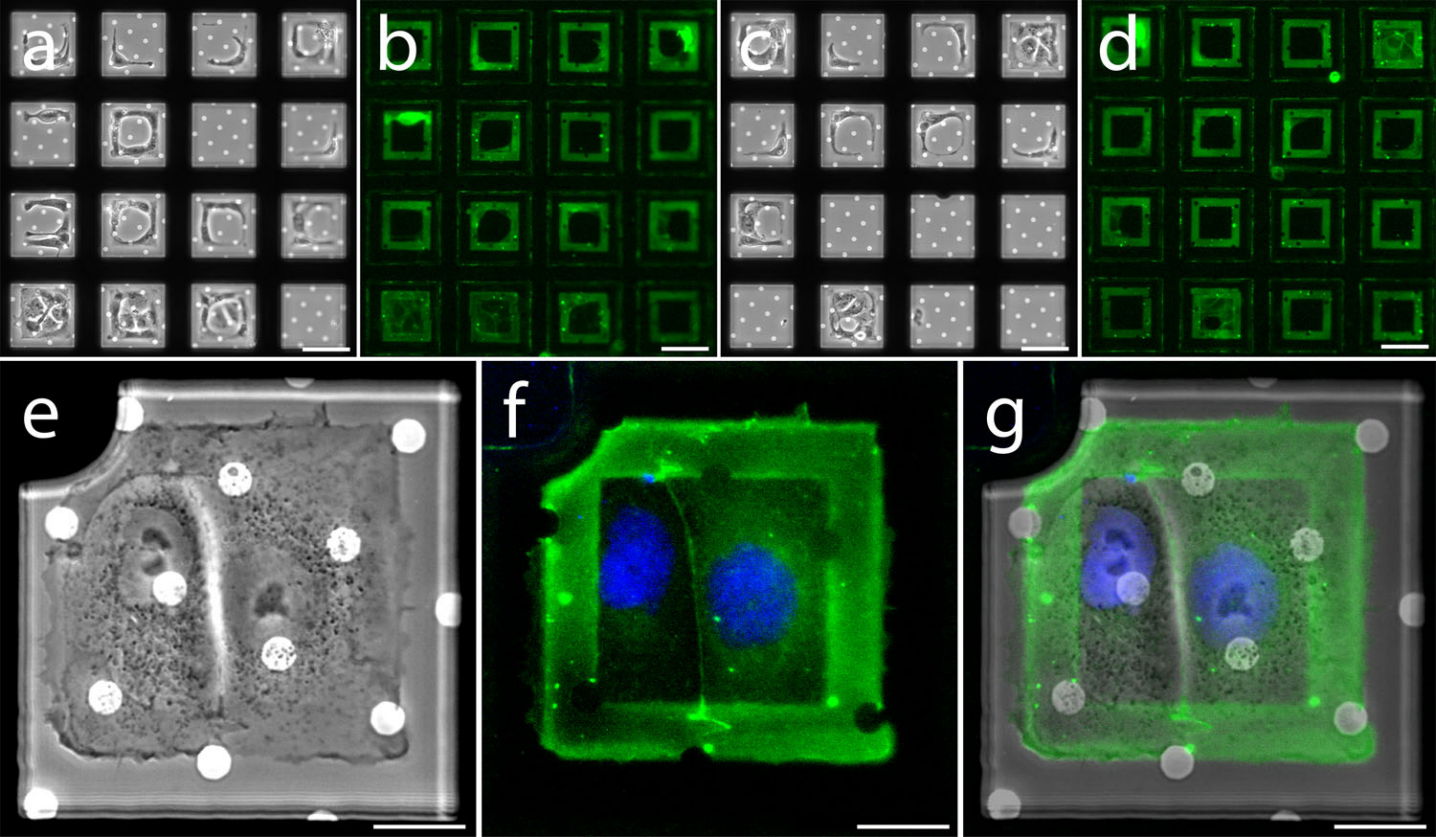
Confinement of epithelial cell pairs on patterned holey carbon electron microscopy grids. Micropatterns are patterned on the carbon film of EM grids and surrounded by a bio-passivated layer to prevent cell adhesion outside of the patterned area, coated with Oregon Green gelatin and then populated with PTK-1 cells expressing EGFP-alpha E catenin. **a**, **c** Phase contrast and **b**, **d** corresponding fluorescence images of patterned areas are shown in the top panels. **e**–**g** Representative cell doublet fully confined within the patterned area (**e**- phase contrast, **f**- fluorescence: DAPI in blue; Oregon Green gelatin and EGFP-alpha E catenin in green, and **g**- overlay of phase contrast and fluorescence images). Scale bars, 60 µm (**a**–**d**) and 20 µm (**e**–**g**). All ECM patterns are 66.5 × 66.5 µm.

To verify confinement of cells on the patterns, we seeded Potorous tridactylus kidney-1 (PTK-1) epithelial cells on carbon coated electron microscopy grids patterned with 66.5 × 66.6 µm gelatin squares (Fig. 4). PTK-1 cells are thinner and flatter than other epithelial cells yet maintain the distinctive features of the adherens junction. Therefore, these cells were ideal for electron tomography of their cell–cell contacts. To be able to validate the maturity of the epithelial cell–cell contacts, we generated a stable cell line in PTK-1 cells expressing an EGFP tagged form of mouse alpha E catenin (Supplementary Fig. 6). The cells were unable to bind to the outer PLL-g-PEG layer and thus spread across the available patterned protein, filling >50% of the available patterns (Fig. 4a–d). The square patterns facilitated a predictable cell geometry and consistent spatial control of cell–cell contacts (Fig. 4e–g). Furthermore, our workflow allows to spatially regulate different cell types with different ECM substrates (Supplementary Figs. 7 and 8). However, to achieve consistent spatial regulation of cell pairs we identified several factors that must be carefully regulated; for example, cell seeding methods must be optimized and the integrity of the bio-passivation layer must be validated (Supplementary Fig. 9). With all parameters optimized, we currently achieve success rates between 27% and 70% (i.e., 30–73% of the patterns are empty, damaged, or overcrowded). About a third (33.4 ± 7.3%) of the patterns with adhered cells had cell–cell contacts clearly identifiable by the EGFP-alpha E catenin signal. With the patterned area of the grid restricted to the central 10 × 10 squares, this percentage results in 9–23 candidates per grid for further analysis.

### Correlative light and cryo-EM/ET visualization of cell–cell contacts

**Fig. 5:**
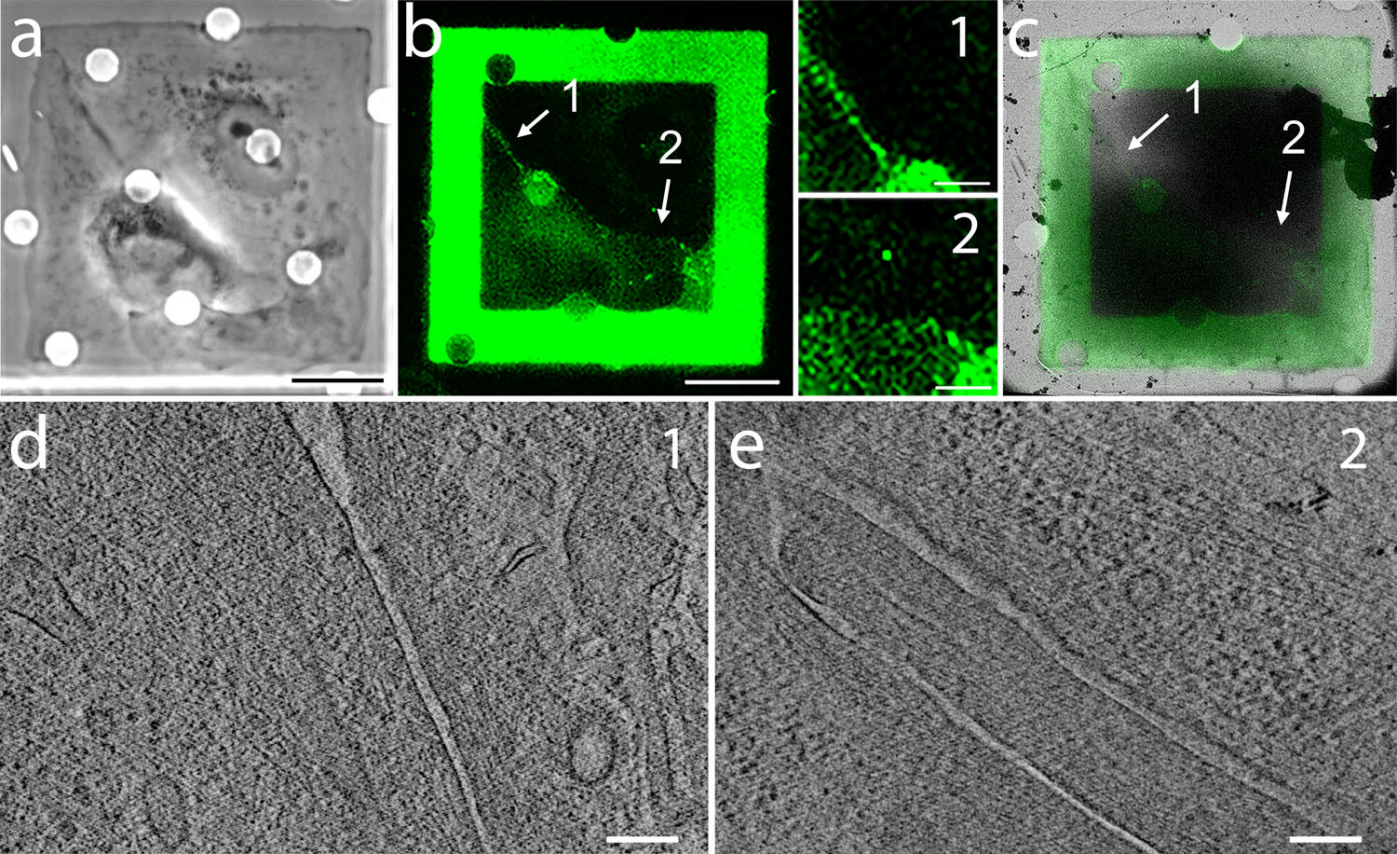
Correlative light and cryo-EM/ET visualization of cell–cell contacts in PTK-1 cells adhering to micropatterns. PTK-1 cells stably expressing EGFP-alpha E catenin were plated on micropatterned grids treated with Oregon Green gelatin. Upon adhesion, many cells form doublets confined into the pattern, as shown in this example. **a** Phase contrast, **b** fluorescence image of the cell pair of interest, displaying both the EGFP signal from the tagged alpha E catenin and the Oregon Green signal marking the pattern. Note that the pattern signal intensity is inherently much higher than that of the tagged protein, somewhat overwhelming the EGFP signal, which is quite robust in the absence of the pattern (see Supplementary Figs. 6 and 8). The numbers “1” and “2” point to two regions rich in cell–cell contacts and thin enough for direct EM imaging. The insets to the right show the two regions at higher magnification and increased contrast. **c** Overlay of the aligned fluorescence and cryo-EM views used for identifying the same two regions selected in **b** for investigation by cryo-ET. **d**, **e** 2-nm thick slices from tomographic reconstructions obtained from regions 1 and 2. Scale bars, 20 µm (**a**–**c**), and 200 nm (**d**, **e**). All ECM patterns are 66.5 × 66.5 µm.

We next performed cryo-ET of spatially regulated cell–cell contacts in confined cell pairs. Since cell–cell contacts are difficult to identify using traditional light microscopy, we used the EGFP – alpha E catenin cell line we had generated in PTK-1 cells to better locate contact sites (Fig. 5a, b). We additionally utilized cryo-CLEM to map how spatial regulation of cell–cell contacts locally altered the total thickness of the sample in the contact region (Fig. 5c). For example, cells that adhere to larger patterns can have thinner (in z) cell–cell contacts^30^. Therefore, we wanted to verify that stretched cells on micropatterns created regions thin enough for imaging. Our initial cryo-ET experiments indicated that while the body of the cell was often too thick for initial cryo-ET, many regions of cell–cell contact sites were thin enough for imaging. In multiple locations along the cell–cell contact, we generated tomographic reconstructions from tilt series with an angular range of ±66˚ (Fig. 5d, e). When the sample is tilted, the grid bars can obscure the field of view if cells of interest grow near the edge of the grid square, even if they are fully visible in the untilted image. Furthermore, the physical edge of the grid, the clip ring that encases it, and the holder/stage itself obscure entire regions radially outward from the physical center of the grid even at lower tilts (Supplementary Fig. 10a). Thus, to increase the efficiency and coverage of tomography data collection, we positioned our micropatterns to confine the cells to regions that allow full tilt range of ±66˚ (Supplementary Fig. 10b). By utilizing the full tilt range, our methods allow recovery of the maximum amount of three-dimensional information.

We were able to verify the integrity of our cells within confining micropatterns by imaging the plasma membrane and major cellular organelles including, mitochondria, vesicles, and actin fibers spanning different regions of the cell (Supplementary Fig. 11). These results demonstrate the capability of cryo-ET to faithfully image spatially regulated cell–cell contacts of healthy intact epithelial cells.

### Cryo-FIB milling of cell–cell contacts

**Fig. 6:**
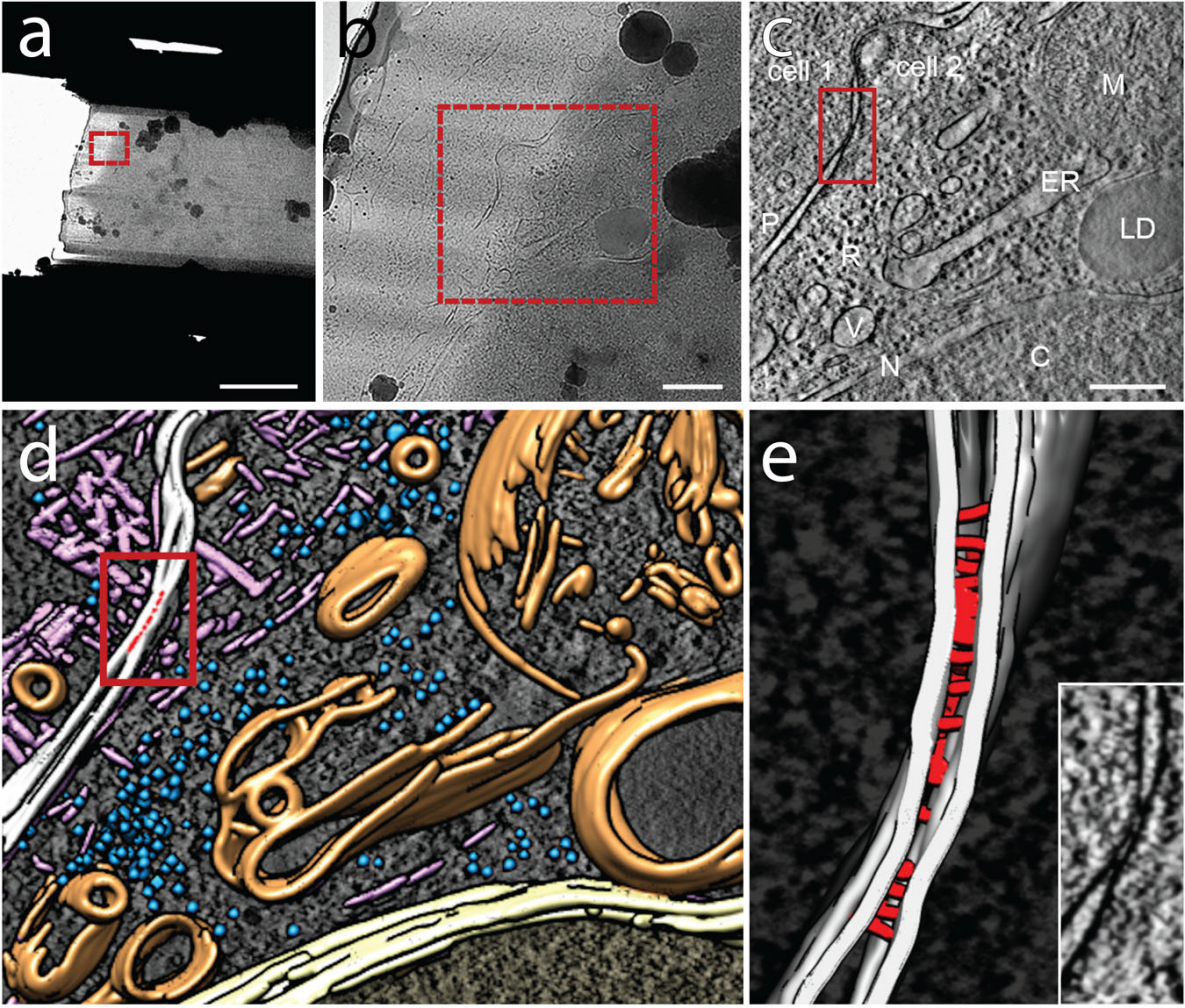
In situ cellular tomography of shape-guided thinned region of micropatterned adhering PTK-1 cell–cell contacts. **a** Top view of a cryo-FIB-milled lamella across a vitrified PTK-1 cell–cell contact site imaged using a cryo-transmission electron microscope. The milling patterns above and below the sample were adjusted to leave a central ~200 nm thin slice (lamella) supported by the surrounding unmilled, vitrified material. The cuts below and above the lamella are for releasing stress introduced by the milling. Cell–cell contact regions are identified visually using the shape of the two interacting cells in cryo-SEM images (Fig. S12a, b illustrate shape-guided targeting of a region of interest). **b** Higher magnification of targeted region marked by the red box in (**a**) representing the area of tomogram acquisition. **c** 2-nm thick virtual slice through a tomogram of the lamella region marked by the red box in **b**, showing a clearly discernible cell–cell contact (marked by red box, P: plasma adjacent plasma membranes) between two adjacent cells. Other cellular features visible include dense packing of macromolecular complexes in the cytoplasm; R: ribosomes; ER: endoplasmic reticulum; M: mitochondrion; V: vesicle; LD: lipid droplet; N: the nuclear envelope separating the nucleoplasm from the cytoplasm and C: Chromatin within the nucleus. **d** 3D surface representations of segmented features. Plasma membranes in white, nuclear envelope in yellow, other membrane structures in orange, ribosomes in blue and actin filament assemblies in pink. The cell–cell contact is marked by a red box. A slice of the tomogram is shown in the background for reference. **e** 3D surface representation of adherens junctional punctum within cell–cell contact. Membrane is in white, connecting rods are in red. The inset shows an enlarged view of the boxed area in **c**. Scale bars in *a* = 5 μm; *b* = 500 nm, *c* = 250 nm.

While we were able to successfully image cell–cell contacts, features of the adherens junction within the cellular interior remained inconclusive. Therefore, we utilized cryo-FIB milling to isolate regions of the cell–cell contact along the basal-apical axis (Supplementary Fig. 12). Prior reports posit the adherens junction in the upper portion of the cell–cell contact along the apical-basal axis, ~500 nm below the apical side of the cell^43^. Therefore, we milled a 200 nm thin slice to isolate a lamella from this region of the cell (Fig. 6a, b). The 3D reconstruction of the imaged area shows intricate cellular details, including the abutting plasma membranes of the two cells (Fig. 6c). Some regions of the plasma membranes are in close contact (red box in Fig. 6c) revealing an isolated punctum reminiscent of punctate adherens junctions observed in conventional thin-section electron micrographs^44,45^. Near this punctum, a high density of actin filaments forming tight bundles and dense networks is visible. Segmentation analysis (Fig. 6c, d) reveals a dense network of irregularly placed rods of 4.5-nm diameter bridging the plasma membranes with an inter-membrane distance of 15.4 ± 3.0 nm at the punctum (Fig. 6e, red). These values are consistent with those obtained from quick-freeze deep-edge microscopy of in situ adherens junctions^45,46^. A fraction of the rods cluster into larger assemblies with an increased width of up to 20.4 nm. The ratio between clustered and individual rods is 1.3, close to the 1.2 ratio between clustered and individual E-cadherin dimers observed in super-resolution light microscopy of adherens junctions^47^.

The height of the lamella in respect to the cell surface should ideally be guided by the fluorescence signal of some adherens junction marker like the EGFP-tagged alpha catenin used in this study. However, technologies that allows accurate enough Z-localization of the fluorescence signal for accurate cryo-FIB application is not currently available. An emerging technology that combines cryogenic fluorescence imaging directly with the cryo-FIB milling step^48^ allows checking the fluorescence signal during milling, offering some potential guidance for lamella production. However, the required hardware is not yet widely available. Nevertheless, the geometric constraints used here generate a surprisingly high yield of junctions and thus offers a viable alternative. We found at least one junction similar to the one depicted in Fig. 6 in 89% of the successful lamellae. In total, we produced 15 lamellae, 9 of which yielded interpretable tomograms. For all but one of these lamellae, at least one of the tomograms generated from the respective lamella contained one or more junctions. Our current protocol yields 9–23 candidates per grid (see above). From our current experience with operating the Aquilos 2 system, 15–20 lamellae can be generated from a single grid sample using semi-automated milling during a standard session constituting two consecutive days. This range is a good match with the expected number of junction candidates per grid.

## Discussion

Cryo-ET has created an imaging revolution in structural biology over the last decade^17,24^, but has been relatively low-throughput and is expensive and labor intensive. In recent years, there has been increased accessibility and interest in using protein micropatterns to regulate cell positioning on EM grids for cryo-ET^30,31,35,49^. While this shift has the potential to enable higher sample throughput, the combination of regulated cell boundaries and an underlying carbon film substrate provides a poorly understood microenvironment for cell biology. Here, we provide a nanometer scale characterization of photomolecularly adsorbed protein patterns, and of the carbon substrate. We interrogated the nanoarchitecture of the protein and bio-passivation layers using multiple imaging modalities including AFM, cryo-tomography and scanning electron microscopy correlated with light microscopy. This EM grid patterning workflow builds on and integrates previous techniques to (i) tailor protein pattern sizes and seeding parameters to facilitate cell doublet formation and optimize the field of view for EM tilt tomography and (ii) enable robust sample transportation for multi-site studies using remote equipment. These advances enable cryo-ET of the cell–cell interface as well as other cellular regions within epithelial doublets or single cells (before and after cryo-FIB). We identified well preserved, main cellular features (i.e., intact plasma membrane, mitochondria, ER, micro and intermediate filament, ribosomes) and captured cellular processes including endocytic events. Not only do these tomograms demonstrate the imaging resolution of our technique, but validate the functionality, vitality, and integrity of epithelial cells imaged in our samples.

Because substrate stiffness affects cellular behavior and junction activity, we investigated the mechanical behavior of the carbon films commonly used in cryo-ET studies. By comparing the effective spring constant extracted from AFM force curve measurements of hydrated carbon films on EM grids to an equivalent stiffness of a hydrogel used for cell culture, we estimate that the resistance of a cell pulling axially on the hydrated carbon film of a 200 mesh EM grid is comparable to pulling axially on a polyacrylamide hydrogel having an elastic modulus of 152–264 kPa. While this modulus would be slightly higher when in-plane traction forces are considered, this comparison places cryo-EM grids used in this study in the range of physiological epithelial tissue such as skin^50,51^ or arterial walls^52^. Thus, stiffness in this range is frequently employed in mechanobiology studies using hydrogels^41,53^. Our measurements provide essential parameters to predict how cells could potentially deform the carbon film in the context of our studies, as well as any future studies using the carbon film as a cell culture substrate.

Observed by standard light microcopy (resolving power >200 nm), protein micropatterning appears to produce straight edges and reproducible patterns. However, we found that at the nanometer scale the edge of the pattern undulates due to the nature of the photopatterning method. Therefore, cells on photopatterned EM grids interact with an edge akin to the shape of a coastline viewed from an airplane, which has implications for incorporating assumptions of smooth boundaries into physical models of force distributions and may influence the localization of integrin binding sites (e.g., clustering of adhesions in nanoscale size pockets of the ECM patterns). Furthermore, the topographical differences between the ECM micropatterns and the encompassing PLL-g-PEG bio-passivation layer indicate that unlabeled ECM proteins can be visualized on the carbon film during electron tomography, facilitating flexible options for ECM proteins used for micropatterns. Our integration of multiple imaging modalities for ECM characterization (e.g., AFM, cryo-tomography, and correlative light and EM microscopies) will support molecular quantitative biophysical predictions.

Our workflow allows us to perform cryo-ET on minimally thick protein patterned samples independently of cryo-FIB. Here, the thickness of the carbon film ranges from 10–12 nm and gelatin patterns add a few nanometers in height, well within the thickness constraints of cryo-tomography (<300 nm). These results serve as a reference for estimating thicknesses of other ECM proteins (e.g., laminin, fibronectin, or collagen) in other cryo-tomography studies. For cellular samples or regions of interest too thick for electron permeation, our workflow incorporates cryo-FIB.

Cryo-FIB is necessary to isolate specific regions of the cell–cell contact to uncover the molecular structure of single cell–cell junctions. cell–cell contacts can be up to several microns thick in the z direction (perpendicular to the grid surface), with a variety of junctions linking the two cells. Furthermore, different junctions can exist within the same z-plane. The punctate protein structure we revealed within the complex epithelial cell–cell contact exemplifies the pristine preservation of nanostructures within our workflow. The morphology at the imaged cell–cell contact is not consistent with the close apposition of membranes in a tight junction nor the cytoskeletal interactions or regular morphology of a desmosome. In fact, the morphology is reminiscent of punctate adherens junctions observed in conventional thin-section electron micrographs^44,45^ a dense network of irregular, rod-like connections between the plasma membranes similar to those observed with quick-freeze deep-edge microscopy of adherens junctions^45,46^. Together, these facts lead us to believe that the imaged region is likely part of a punctate adherens junction. Future work will focus on exploring labeling methods for specific junctions under cryo-ET, allowing us to validate specific cell–cell junctions and elucidate their changes in molecular structure.

Our ability to segment single junctional structures within cell–cell contact sites serves as a significant innovation in determining the relationship between cell–cell communication and cell behavior. Future studies could therefore focus on how changes on the cellular microenvironment (e.g., ECM composition or cell shape) influence cell–cell coordination (e.g., protein localization, actin bundling, junction formation). Beyond epithelial studies, other mechanobiological systems can implement our workflow to determine the fundamental structural changes governing cell–cell signaling. Our workflow not only can isolate these cell–cell contacts for study in the observable regions of an EM grid, but our implementation of cryo-FIB can isolate any region along the contact zone for detailed cryo-ET. Ultimately, this research paves the way for an enhanced understanding of (i) how the cellular microenvironment regulates molecular changes at cell–cell contacts, and (ii) how changes in epithelial contacts regulates aspects of cellular function.

## Methods

### Grid preparation and handling

200-mesh R5/20 holey-carbon coated gold grids (Quantifoil Micro Tools GMbH) were first inspected for potential carbon tears and only grids with minimal damage were used. Grids were then placed carbon side up on 250 µm-thick 5 mm-diameter PDMS platforms (B&J Rubber Products, Bishop, CA, USA) cut from a Silhouette CAMEO 3 electronic desktop cutter (Silhouette America, Inc., UT, USA). Adhered on a #1.5 glass coverslip, the samples were plasma cleaned for 5 min at 18 W (Harrick, PDC-32G). Immediately after plasma treatment, a 600 µm-thick PDMS ring (8 mm inner diameter, 12 mm outer diameter) was sealed around the grid (Supplementary Fig. 1). A solution of 100 µg/mL of poly(l-lysine)-graft-poly(ethylene glycol) (PLL(20)-g[3.5]- PEG(2)) (SuSoS AG, Dübendorf, Switzerland) diluted in phosphate buffered saline (PBS) was immediately placed within the well and incubated for 1 h at room temperature. The well was then rinsed thoroughly (10x) with PBS prior to micropatterning. Following micropatterning, a 12 mm glass coverslip was adhered to the outer PDMS ring to seal in buffer for transport and additional handling.

### Photopatterning and protein functionalization of EM grids

Following PLL-g-PEG incubation, excess PBS was removed from the well containing the grid, with enough remaining to keep the sample hydrated. 15 µL of UV sensitive photo initiator (PLPP, Alvéole, Paris, France) was added to the well and the sample was placed on the stage of a Leica DMi8 epifluorescence microscope equipped with a Fluotar 20x/0.40 N.A. objective and the Alvéole Primo photopatterning system (Alvéole, Paris, France). Digital masks for ECM patterns were made using the open-source software program Inkscape^54^, which were loaded onto the Leonardo plugin (Alvéole Laboratory) on μManager software^55^. The pixel/µm ratio generated by Primo calibration was used to define the geometries of ECM patterns. For each grid, a single mask consisting of a 10 × 10 array of hollow square micropatterns (50 × 50 µm or 66.5 × 66.5 µm, 8 µm thickness) were angled to the center the grid squares, then projected through the carbon film at a dosage of 2500 mJ/mm^2^ from a 375 nm, 7.10 mW laser. 35 × 35 µm hollow square micropatterns were used for MDCK cells (Supplementary Fig. 8). Since the digital masks were slightly larger than the DMD, imperfect DMD stitching between masks during patterning created subtle misalignments in several patterns (Supplementary Fig. 2). Alternative approaches can be used to mitigate such stitching errors. Therefore, subsequent protocols used smaller 2 × 3 array masks. Following micropatterning, the photo initiator was rinsed off with PBS and a 100 µg/mL solution of Oregon Green gelatin (ThermoFisher, G13186) was incubated on the grid for 1 h at room temperature in the dark. The grids were then rinsed thoroughly with PBS and stored hydrated at 4 °C prior to cell seeding.

For functionalization with rhodamine fibronectin, EM grids were prepared using an overnight incubation 0.01% poly-l-lysine (Sigma) in a humid chamber at room temperature, washed 3x with HEPES 0.1 M pH 8.4, and incubated for 1 h with freshly prepared 100 mg/mL polyethylene glycol-succinimidyl valeric acid (PEG-SVA) resuspended in HEPES 0.1 M pH 8.4. Photoscission of the PEG-SVA chain was performed using a laser dosage of 1200 mJ/mm^2^ and a 4.5 mW laser power. Grids were then washed in 500 µL of water and 2 times in PBS. Micropatterned grids were functionalized by incubation for 1 h with 20 µL of 50 µg/mL of Fibronectin-Rhodamine (Cytoskeleton) and washed 3x in PBS.

### Atomic force microscopy

#### Pattern characterization

The thickness of the PLL-g-PEG and the gelatin layers was measured using the BioScope Resolve AFM (Bruker) coupled with the Axio-Observer inverted fluorescence microscope (Zeiss). The AFM topography was measured in PBS solution at room temperature (~22 °C) using PeakForce mode. The AFM probe was a MLCT silicon nitride cantilever (*Bruker*) with a spring constant of ~0.1 N/m, as reported by the manufacturer. The image was processed using Nanoscope Analysis 2.0 software (Bruker). For fluorescence imaging, the Oregon Green filter-set (excitation 488 nm, emission 525 nm) was used.

#### Carbon film force deflection

AFM loading measurements were performed with a WITec AFM (Alpha300) and a 200-mesh R 5/20 holey-carbon coated gold grid (Quantifoil Micro Tools GMbH). The grid was submerged in PBS and adhered to a strip of PDMS to prevent slippage. We used a cantilever probe with a tip-radius curvature of 1 µm and nominal spring constant, *k*_*c*_, of 0.251 N/m (SAA-SPH-1UM, *Bruker*). Optical lever sensitivity of the cantilever probe was measured by performing force scans against a glass surface prior to carbon film measurements and was found to be 25.97 nm/V. For each measurement, the tip of the AFM cantilever was centered above the grid square before approaching the surface. The following feedback threshold parameters were used to locate the surface: 1.0 V set point (25.97 nm cantilever deflection), 1% p-gain, and 0.2% i-gain. To avoid potential hysteresis effects, each square was loaded only once. Each loading measurement was performed using WITec’s Distance Curve mode with approach and retract distance settings of 100 nm (total vertical travel of 200 nm) at speeds of 100 and 1000 nm/s. The deformation of the carbon film was determined by taking the difference between the piezo position and the cantilever deflection (Supplementary Fig. 4)^56–58^. The spring constant from each measurement was calculated by using total least squares regression between the force applied by the cantilever and the deflection of the carbon film. We observed periodic noise in the measurement introduced by ambient noise and vibration. Based on a Fast Fourier Transform analysis of the force curves (Supplementary Fig. 5), the main sources for periodic background noise were ~60 Hz and ~300 Hz, which match the 1^st^ and 5^th^ harmonics of the electrical systems in the lab. Statistical significance between data sets was determined using a two-tailed student t-test assuming equal variance.

### Generation of new cell line stably expressing EGFP-alpha E catenin

PTK-1 cells (source: ATCC CRL-6493) in log phase were electroporated with pEGFP-C1-ACAT (mouse alpha catenin subcloned into pEGFP-C1, ClonTech; EGFP is fused to the carboxyl terminus of the target protein; construct was a generous gift from Nelson’s group). After selection with G418 (400 µg/mL), resistant cells went through three rounds of enrichment by sorting. Once a bulk population and single cell clones were isolated and grown, they went through testing by western blot (WB) and immunofluorescence (IF). WB was performed using cell equivalent lysates (the same number of cells was used for each lysate) from parental, bulk, single cell clones and a control stable line generated with the same construct in MDCK cells (source: parental MDCK.2, ATCC CRL-2936). IF was conducted according to standard protocols with a dilution of 1 µg/ml. Both WB and IF were done using an antibody against alpha catenin (mouse monoclonal antibody against alpha catenin 15D9, AM00014PU-N, OriGene Technologies Inc.; kindly provided by Nelson’s group).

### Preparation of micropatterned grids, cell plating, and titration for cryo-ET

Micropatterned grids were rinsed once in sterile 1x PBS and screened for integrity and quality of patterns on an inverted light microscope (Eclipse TE 2000-U, Nikon) equipped with manual controlled shutter, filter wheels, and a 14 bit cooled CCD camera (Orca II) controlled by MetaMorph software (Universal Imaging), using a Plan Fluor ELWD 40/0.60 Ph2 or a Plan Fluor 10/0.30 Ph1 objective lens (Nikon). Alternatively, micropatterned grids were imaged using a Spinning Disk (CSUX M1-L, Solamere) attached to a standard fluorescence widefield inverted microscope (Axio Observer 7, Zeiss). Grids were removed from PBS, equilibrated in complete medium for approximately ten minutes and seeded with cells in log phase. As our goal was to obtain doublets in each pattern, for as many patterns as possible, the cell density was titrated by applying 10,000–100,00 cells/mL in increments of 10,000 cells. The titrations indicated that a cell concentration around 50,000 cells/mL was ideal in obtaining a good percentage of doublets in each pattern over the course of a 12-hour incubation. Once cells had been seeded, those that had not adhered within the first two hours were removed by gentle rinsing with complete medium and then continued the course of the incubation. Upon completion of the 12-hour course, cells were fixed in a buffer composition determined in the RCIA protocol^59^ where the medium was removed and replaced with a fixative solution (4% PFA diluted in minimal formulation of PHEM (0.1 M Pipes, 1 mM EGTA and 1 mM MgSo4). The initial 5 min were at room temperature, while the remaining 25 min were carried out over ice. After 30 min of incubation, the fixative solution was removed, and grids were gently rinsed three times in cold complete PHEM buffer (60 mM Pipes, 25 mM HEPES, 2 mM MgSo_4_, 10 mM EGTA, pH 6.9)^59,60^. The light microscopy information, collected on the inverted microscope set up described above was used to improve the precision of selecting regions of interest for cryo-tomography data collection. For observation, each grid was flipped (cells face down) and placed in a MatTek dish in presence of PHEM. Areas of interest were identified, their positions recorded and phase contrast, fluorescence images were collected. Upon completion of this survey, grids were plunge frozen.

### Vitrification and cryo-EM screening

Prior to vitrification, 1–2 µL of fiducial markers were added to the samples (Gold Colloid 15 nm, BBI Solutions). Vitrification was performed using a home-designed cryo-plunger; fixed and surveyed samples were manually plunge-frozen in liquid-nitrogen-cooled liquefied ethane and stored in liquid nitrogen until investigation. Freezing quality, along with assessment of sample preservation and amenability for cryo-ET investigation was conducted by an initial screening on a T12 Spirit (ThermoFisher) cryogenic transmission electron microscope (cryo-TEM) operated at 120 keV with an Eagle CMOS detector (ThermoFisher) or a Glacios (ThermoFisher) cryo-TEM operated at 200 keV with a Falcon 3EC direct electron detector. Using SerialEM^61^ we generated a montage for each cryo-grid and identified target cells for either direct imaging or cryo-FIB milling by following the finder markings on the grids. The fluorescence images of patterns and cells on patterns collected after fixation and before vitrification, were precisely aligned with images of the same targets obtained by cryo-EM, using a fiducial-less approach^19^.

### Cryo-FIB Lamella preparation

Grids were clipped using special Autogrids containing a milling slot (ThermoFisher). The milling slot allowed the access of the gallium beam at shallow angles appropriate for the cryo-lamellae preparation. Autogrids were transferred to the Aquilos 2 (ThermoFisher) dual beam cryo-FIB instrument. Cryogenic lamellae sites were chosen based on the cell–cell contact area observed by the scanning electron microscope (SEM) beam of the Aquilos 2 and correlated with the imaging obtained from the DM6 cryo-Light microscope (Leica Microsystems). Fluorescence images were aligned with the SEM tile set file using MAPS 3.14 software (ThermoFisher). A total of 15 cryo-lamellae were prepared using the semi-automated software AutoTEM 2.2.0 (ThermoFisher) for rough milling and the manual interface (XT User v20.1.0 ThermoFisher Scientific) for lamellae thinning and polishing with a final thickness below 300 nm. Autogrids were coated with a protective organometallic platinum layer by the gas injection system (GIS) of the Aquilos 2 for 12 s. Rectangular masks of 10 µm to 20 µm height were used for thinning and polishing steps of lamellae preparation at different currents using the gallium focused ion beam of the Aquilos 2. The rough milling step was performed at 0.5 nA ion beam current to generate a 2.5 µm thick lamella. The subsequent milling was performed at 0.3 nA ion beam current, thinning the lamellae to a thickness of 1.5 µm. The next step was performed at 0.1 nA current down to a lamella thickness of 1 µm and finally a current of 50 pA thinned the lamellae down to 500 nm. The fine polishing was performed at 30 pA ion beam current, resulting in a final thickness of 200–300 nm. Cells, patterns, and cryo-lamellae were imaged by SEM at 2 kV to 5 kV.

### Correlative light and cryo-EM, cellular cryo-tomography

Electron cryo-tomography data were collected using SerialEM^61^ or the Tomo package (ThermoFisher) on Titan Krios (Thermo Fisher) or Glacios cryo-microscopes. The Titan Krios used for direct imaging was equipped with a Falcon 3EC direct electron detector (Thermo Fisher), the Titan Krios used for imaging lamellae was equipped with a K3 direct electron detector with a BioQuantum energy filter (Gatan Inc.) with energy slit set to 20 eV and using 70 µm condenser aperture and 100 µm objective aperture. Both Titans were operated at 300 keV. The Glacios microscope (ThermoFisher) used for some of the lamellae was operated at 200 keV and was equipped with a Falcon 3EC direct electron detector (ThermoFisher) and using 50 μm condenser aperture and 100 μm objective aperture. The autogrids were rotated 90° between Aquilos-2 and Glacios use to accommodate the tilt-axis orientation in the Glacios TEM. All three instruments were equipped with extra-bright Field Electron Guns (XFEG) and were operated in parallel beam condition. For each grid, an overview image (atlas) of the entire grid was acquired to locate lamellae or pre-screened cells for direct imaging. Markings on the Finder grids, which are visible in light microscopy and cryo-EM/ET imaging modalities were used for guidance. Low-resolution images of the grid squares (pixel size 14–28 nm) containing the identified cells or lamellae enabled alignment of light microscopy and cryo-tomography images of the same cell. Following correlative identification of regions of interest were applicable, dose-symmetric tilt series (± 60˚, every 3˚) were collected in batch mode under minimal dose conditions of about 90–120 e^-^/Å^2^ and defocus between 8 and 14 μm with magnification resulting in a calibrated pixel size of 0.48 nm.

### Tomographic reconstruction, volume processing, and segmentation

Tomographic reconstructions were calculated automatically during data collection as implemented in the pyCoAn package (github.com/pyCoAn/distro), an extended python version of the CoAn package. Briefly, immediately after acquisition, tilt series were automatically aligned and then reconstructed using the Simultaneous Iterative reconstruction technique. Alignment and reconstruction statistics were used to determine quality scores that are provided in real time during data collection. All interpretable reconstructions were enhanced using a Wiener-like filter accounting for the contrast transfer function at the respective defocus and an estimate of the spectral signal-to-noise ratio^62^. Segmentation of the features in the reconstructions was achieved using manual tracing of membranes with Amira^63^ combined with fully automated and semiautomatic^64,65^ segmentation approaches as implemented in pyCoAn. Images of tomogram slices were generated with IMOD^66^, images of segmentation models were generated with UCSF Chimera^67^.

### Optimization of imageable regions on 200-mesh Quantifoil copper/gold grid squares using micropatterning

#### Determining the micropattern region to accommodate restrictions of the full grid

To find the regions obstructed by the rim of the grid itself when tilted, we oriented the grids such that the grid bars formed a 45^o^ angle with the tilt axis, the orientation where the maximum amount of grid squares will be potentially obscured by tilting. We used Quantifoil R5/20 Holey Carbon 200 mesh gold grids. Eucentric height was determined and refined at the grid center, then the grid was rotated so that the grid bars and tilt axis formed a 45˚ angle, and tilted to +66˚, the stage was moved along the X-axis and the Y-axis until the beam was obstructed. At these limits the grid was returned to 0˚ tilt and the location on the grid was recorded. This process was repeated after tilting to −66˚ to account for potential asymmetries in the setup. We found that the mesh squares outside of the central 10 × 10 region of squares were potentially obstructed when tilting (Supplementary Fig. 10). Thus, we only patterned the central 10 × 10 squares of the grid.

#### Setting up the micropattern position within an individual grid square

For an individual square to be tested, the grid was rotated so that the grid bars and the tilt axis formed a 45˚ angle. Eucentric height was determined and refined at the center of the square. For each corner along the X and Y-axes, the stage was tilted to +66˚, and then the stage was moved until the reticle marking the center of the detector within the viewing software was positioned in the corner of the grid square to be tested. The stage was then tilted to −66˚ and if the reticle was obscured, the stage was moved until it was visible again. The stage was then returned to 0˚ tilt, and an image was collected. In each of the 0˚ images the distance from the reticle to the two adjacent grid bars was measured. The greatest distance, 18 µm, defines the limit of the pattern size on a 200-mesh grid that allows unobstructed tilt series collection up to ±66˚. For added tolerance, we use 20 µm as safety distance from the grid bars.

### Statistics and reproducibility

Before optimization of the patterning procedure, frequent pattern misalignments occurred (representative images in Supplementary Fig. 2) and cell seeding was often suboptimal (representative images in Supplemental Fig. 9). After optimization, patterning has essentially become routine in the lab and yields consistently well-aligned, well-defined patterns with good cell distributions independent of the pattern shape or patterning agent (representative images in Figs. 1d, 3a–d, 4, and Supplementary Figs. 3, 7, and 8c, d). With all parameters optimized, currently 30–73% of the patterns are empty, damaged, or overcrowded (n = 4 grids). Thus, we achieve success rates between 27% and 70% for patterns with adhered cells useable within the remainder of the workflow. About a third (33.4 ± 7.3%, n = 4 grids) of these patterns have identifiable cell–cell contacts (representative images in Fig. 4e-g and 5a–c), resulting in 9–23 candidates per grid for further analysis. For cryo-ET imaging of empty patterns, 9 tomograms were acquired and analyzed with similar results (representative image in Fig. 2g). For direct cryo-ET imaging of cell pairs (no milling), 31 tomograms from 7 cell pairs were acquired and analyzed (representative images in Fig. 5 and Supplementary Fig. 11). 15 lamellae from 14 cell pairs were prepared, and 26 tomograms were acquired and analyzed from these lamellae. 15 of these tomograms originating from 9 lamellae showed interpretable features (representative images in Fig. 6 and Supplementary Fig. 12). We found at least one junction similar to the one depicted in Fig. 6 in 89% of the successful lamellae.

### Reporting summary

Further information on research design is available in the Nature Portfolio Reporting Summary linked to this article.

## Supplementary information

Supplementary Information

Reporting Summary

## Data Availability

The data that support this study are available from the corresponding authors upon reasonable request. Tomography data are deposited at the Electron Microscopy Data Bank (EMDB) under accession code EMD-15394 (adherens junctional puncta), and the Electron Microscopy Public Image Archive (EMPIAR) under accession code EMPIAR-11114.
