# Supplementary material for: Morphological control enables nanometer-scale dissection of cell-cell signaling complexes
Source: Nat Commun. 2022 Dec 20;13:7831. doi: 10.1038/s41467-022-35409-9 (PMC9768166; doi:10.1038/s41467-022-35409-9)
Supplement: Supplementary file 1 — Supplementary Information [file 41467_2022_35409_MOESM1_ESM.pdf]

Supplementary information for:

Morphological control enables nanometer-scale dissection of cell-cell signaling complexes

Liam P Dow[1], Guido Gaietta[2], Yair Kaufman[1], Mark F Swift[2], Moara Lemos[3], Kerry Lane[1], Matthew Hopcroft[1], Armel Bezault[3], Cécile Sauvanet[3], Niels Volkmann[2,4,*], Beth L Pruitt[1,*], and Dorit Hanein[2,3,†,*]

[1] Mechanical Engineering and Biomolecular Science and Engineering, University of California, Santa Barbara, California, USA
[2] Scintillon Institute, San Diego, California, USA
[3] Institut Pasteur, CNRS UMR3528, Structural Studies of Macromolecular Machines in Cellulo Unit, F-75015 Paris, France
[4] Institut Pasteur, Université de Paris, CNRS UMR3528, Structural Image Analysis Unit, F-75015 Paris, France

[†] Current address: Department of Chemistry and Biochemistry, and of Biomedical Engineering, University of California, Santa Barbara, CA, USA

[*] Corresponding authors: niels.volkmann@pasteur.fr, blp@ucsb.edu, dorit@ucsb.edu

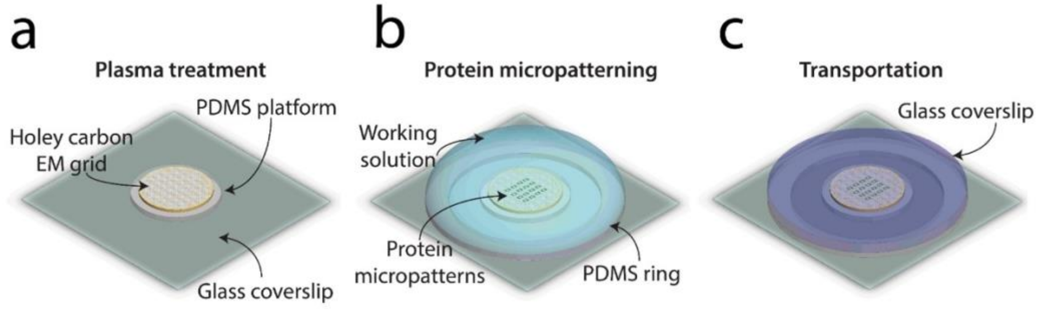

Supplementary Figure S1. Protocol for grid handling and transportation. (a) A thin PDMS platform (250 µm) adheres the metal mesh of the grid, allowing the holey carbon mesh of the topside to be exposed to plasma. (b) A thicker (600 µm), 12 mm diameter PDMS ring is added around the grid following plasma treatment to create a cohesive well for protein patterning, buffer exchange, and (c) a protective and robust seal using a 12 mm glass coverslip.

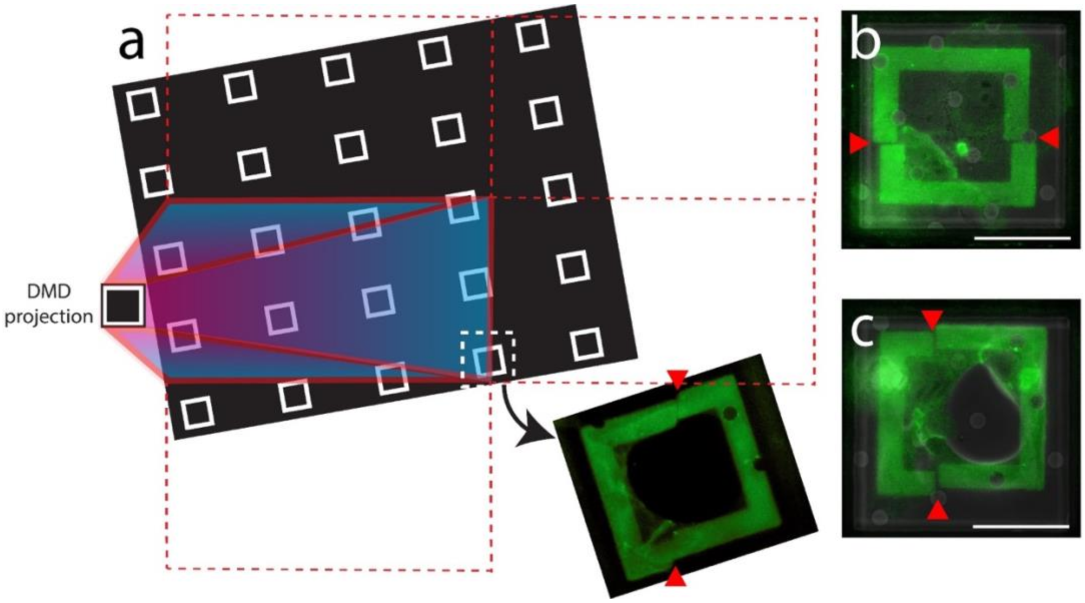

Supplementary Figure S2. Stitching of DMD projections cause pattern misalignment. (a) Calibrated DMD size was 1824 px by 1140 px (approximately 506 µm by 316 µm). Using a 10 x 10 digital mask of individual patterns covering a larger area, the stitching of several DMDs resulted in several misalignments in our initial single patterns. DMD projection zones are indicated by dashed red boxes. (b, c) Misalignment errors can be mitigated using digital masks for single patterns (see Alveole Experiment Wizard) or confining arrays of patterns to single DMDs (e.g., a 4 x 4 array). Scale bars are 30 µm.

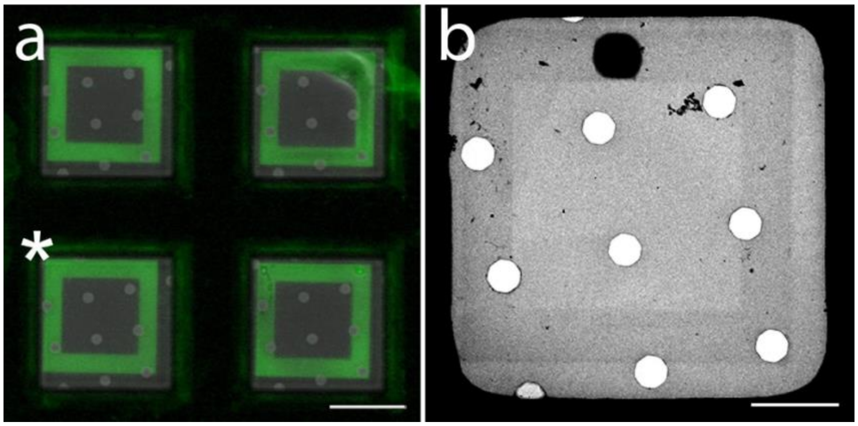

Supplementary Figure S3. **Pattern visualization**. (a) Fluorescence and (B) cryo-EM images of representative micropatterned regions. The pattern marked with a white asterisk in a is shown in b after vitrification of the sample. The pattern is visible in the projection image shown in b due to the density differences between pattern and bio-passivated area. Scale bars, 40 µm (a), 20 µm (b). ECM patterns are 66.5 x 66.5 µm.

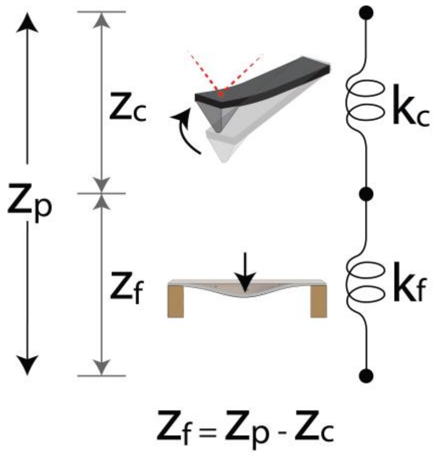

Supplementary Figure S4. Dual spring model of carbon film under load. As the piezo stage moves (z_p) toward the carbon film, both the cantilever and carbon film are deflected elastically (z_c and z_f with spring constants k_c and k_f, respectively). We calculate the z_f using this dual spring model as the difference in displacement of the cantilever deflection from the piezo stage.

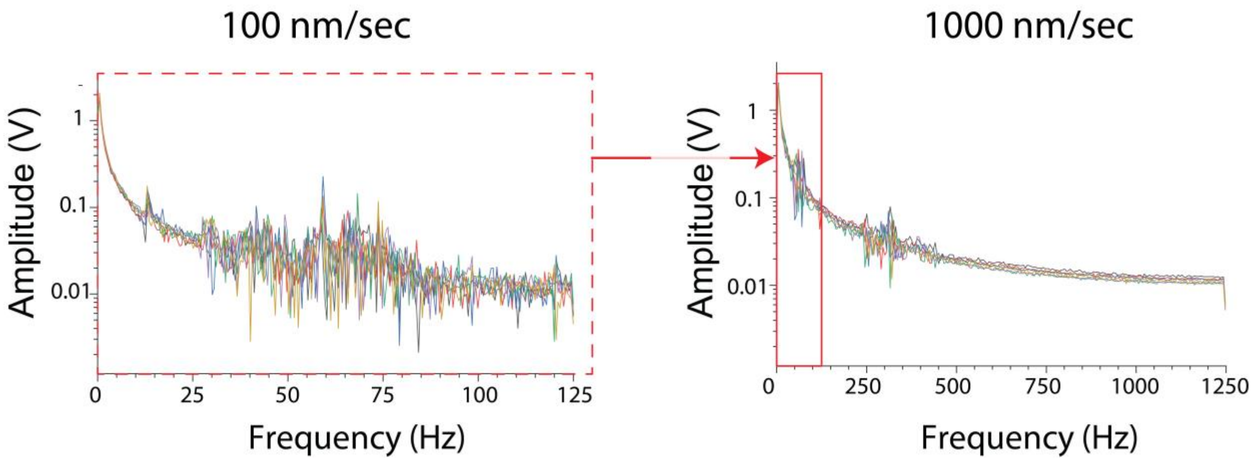

Supplementary Figure S5. Fast Fourier Transform (FFT) of AFM Measurements. AFM measurements were performed at loading rates of 100 and 1000 nm/s (n=6 for both, each n represented as a different color). The AFM data sample rate and measurement bandwidth were proportional to loading rate (e.g. there is no frequency data above 125 Hz accessible for the 100 nm/s loading rate). The red rectangle in the 1000 nm/s graph denotes the equivalent range accessible for the 100 nm/s loading rate (dashed red rectangle). Most distinct noise sources occurred at two locations on the frequency spectra: ~60 Hz and ~300 Hz. These same sources were present for both loading rates, suggesting that the noise was not a function of the measurement procedure.

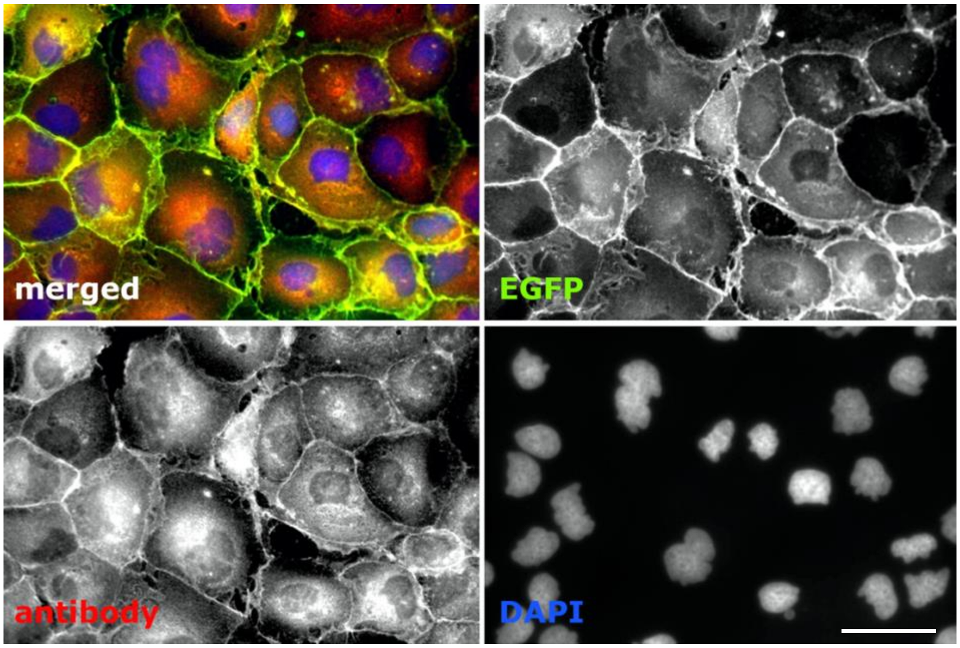

Supplementary Figure S6. **Characterization by immunofluorescence** of the stable cell line used in this study comparing expression and localization of the EGFP-tagged alpha-E catenin (EGFP) with the native form of alpha E catenin expressed by PTK1 cells (antibody). A representative image from three repeats is shown. Cells were allowed to adhere and form cell-cell contacts for 12 hours before fixation. The expression of the tagged protein is homogeneous, its signal for cell-cell contacts is strong and well defined, and its localization overlaps well with the native protein. Scale bar = 25 µm.

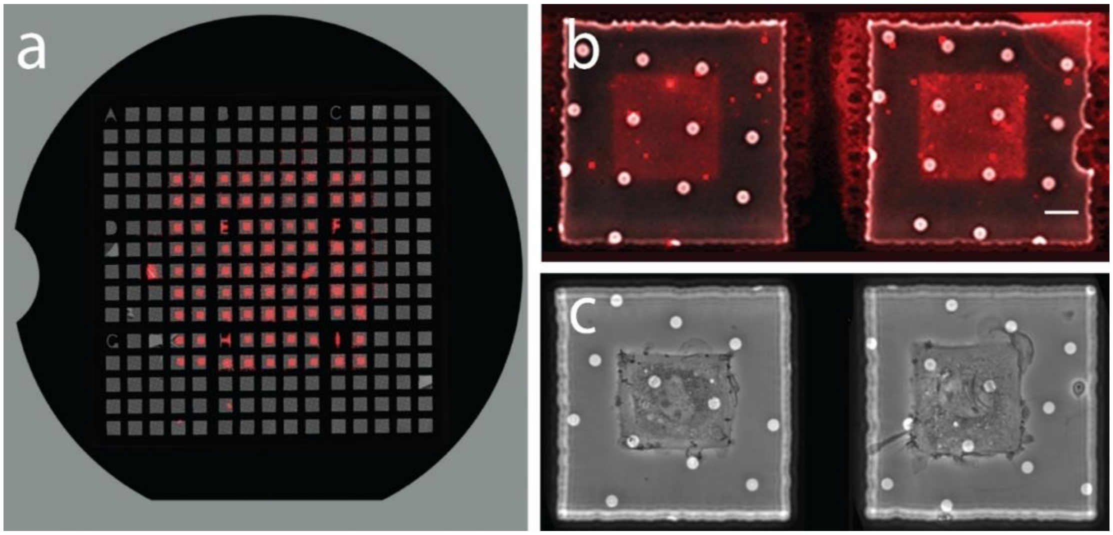

Supplementary Figure S7. **Workflow is agnostic to both ECM and cell type**. (a) Phase contrast light microscopy image of entire grid area overlaid with the fluorescence image of photopatterned Rhodamine-labeled Fibronectin. The 45 µm square patterns are surrounded by bio-passivation made on the holey carbon film of the grid. The use of different ECM protein and fluorescence label, and different patterning mode from Figures 4 and S3, exemplifying the versatility of the patterning approach and ease of incorporating into the workflow. (b) Representative rhodamine fluorescence images overlaid with their corresponding phase contrast light microscopy images showing the fibronectin patterns within the grid squares. (c) Phase contrast light microscopy images of patterned areas with MEF cells adopting the shape of the pattern and being confined within the patterned areas. Diameter of the grid in (a) is 3.05 mm. Scale bar in (b), also applying to (c) = 15 µm.

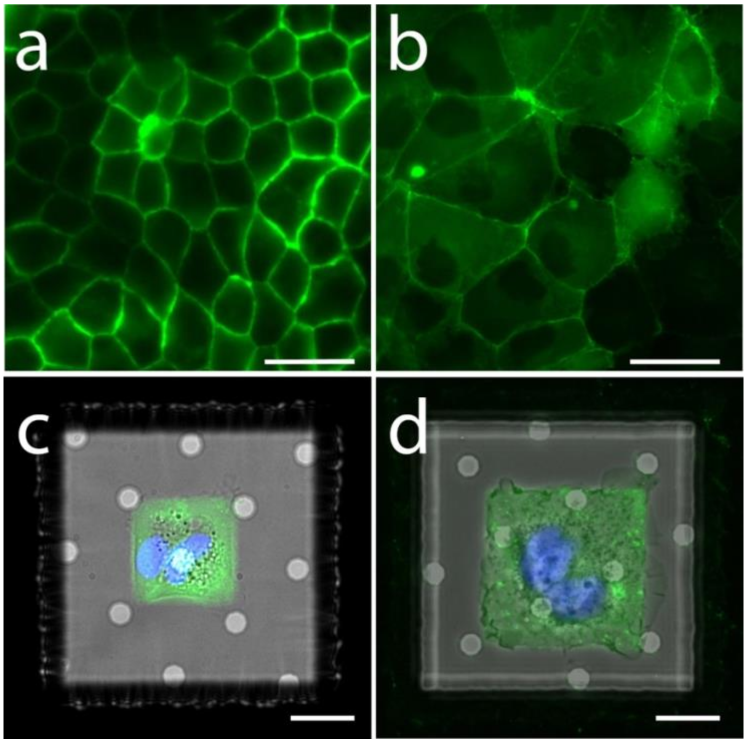

Supplementary Figure S8. Micropattern sizing can be tuned to isolate cell pairs of different cell types. (a) Confluent MDCK cells (E cadherin, GFP) are on average smaller in area than (b) confluent PTK-1 cells (alpha E catenin EGFP). Both images are representative for three repeat experiments each. (c) 35 x 35 µm ECM squares successfully adhere MDCK cell pairs, while (d) larger 50 x 50 µm ECM squares successfully adhere PTK-1 cell pairs. Scale bars are 25 µm (a-b) and 20 µm (c-d). Note that the pattern's Oregon green signal intensity is much higher than that of the tagged proteins. Consequently, the protein fluorescence signals appear diminished in c and d as compared to a and b owing to adjustment of the fluorescence levels when capturing the image.

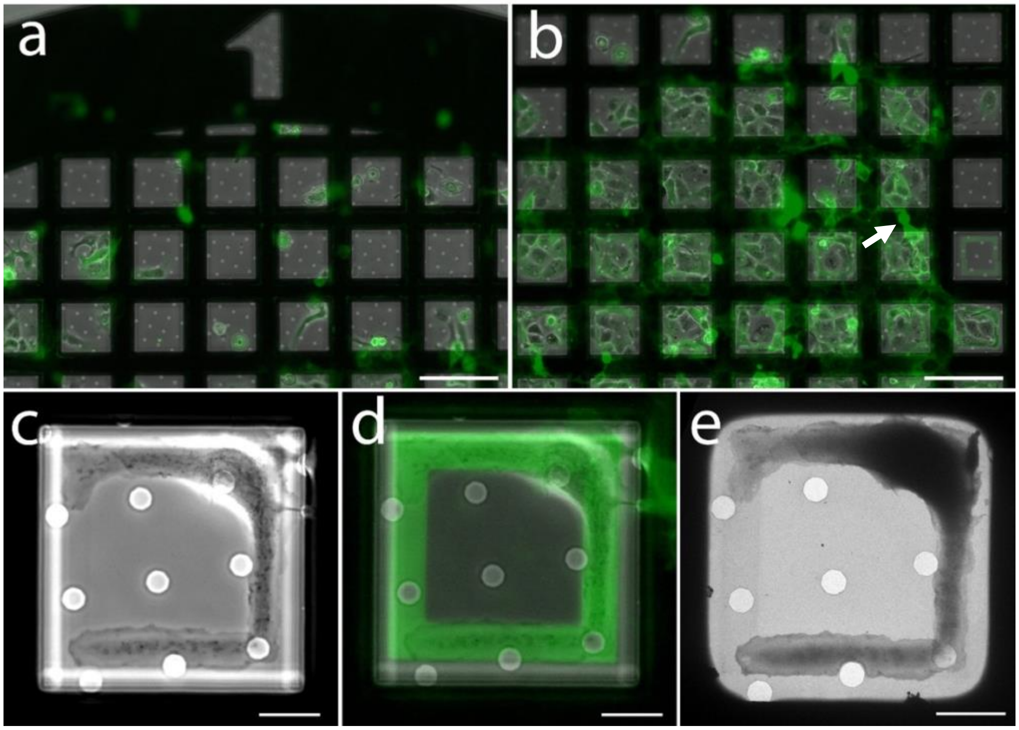

Supplementary Figure S9. Optimization of pattern sizing and cell seeding parameters. Examples of (a) incomplete bio-passivation, (b) excessive cell density at plating and (c-e) undesired cell positioning on the pattern. (a) Incomplete bio-passivation leads to cell adhesion outside of patterns, while high cell density along with unresolved cell clustering result in non-specific adhesion of large number of cells both on patterns and outside of patterns, as show in b. Large clusters of cells are also capable of adhering to neighboring patterns, forming a bridge in between them (white arrow in b). (c-e) An individual cell is shown to spread exclusively on the patterned area, while avoiding the bio-passivated regions. (c) Phase contrast, (d) overlay of fluorescence (Oregon Green gelatin and EGFP- alpha E catenin) and phase contrast images and (e) corresponding cryo-EM image. Scale bars, 100 µm (a, b) and 20 µm (c-e).

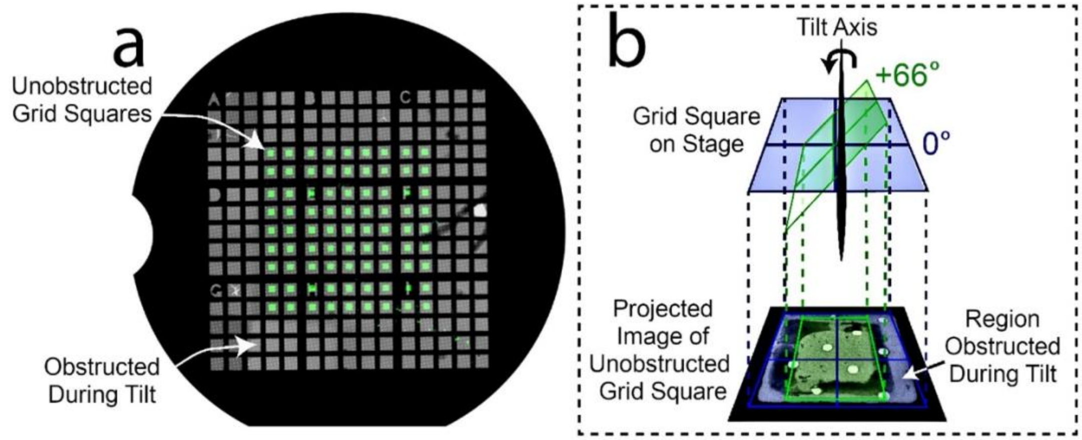

Supplementary Figure S10. Determination of optimal regions on 200-mesh EM grid for tomography data collection. (a) The central 10 x 10 region of grid squares is unobstructed for ±66º tilt range and is patterned (here with Oregon-green labeled gelatin). The region outwards from this central region is potentially obstructed physically by the rim of the grid, the microscope sample holder, or the clip ring and is not patterned. (b) During tilting, an approximately 20-micron wide area on either side of the imageable region of Individual grid squares becomes obstructed by the non-transparent grid bars.

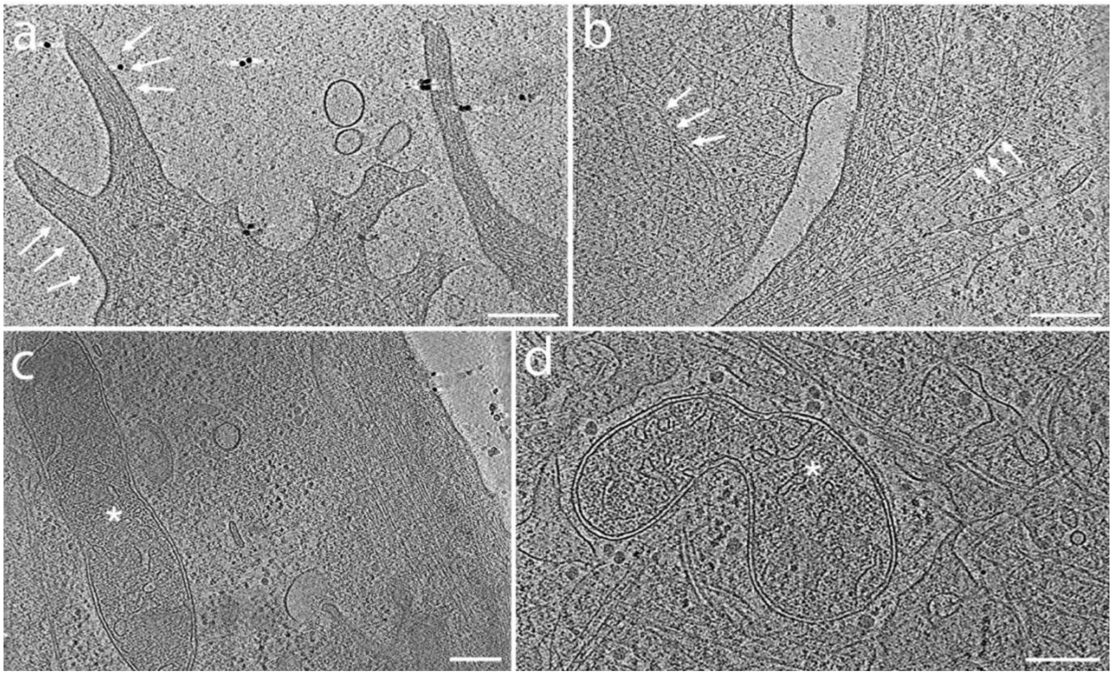

Supplementary Figure S11. Vitrification prevents structural collapse and keeps the sample's ultrastructure close to its native state. Following fixation, treatment and plunge freezing with the suggested protocol, vitrified samples are investigated by cryo-ET. Single slices from representative tomograms targeting (a) plasma membrane regions (white arrows), (b) actin filaments (white arrows) and (c, d) mitochondria (white asterisk) display excellent preservation of the cellular nanoarchitecture. Scale bars, 200 nm

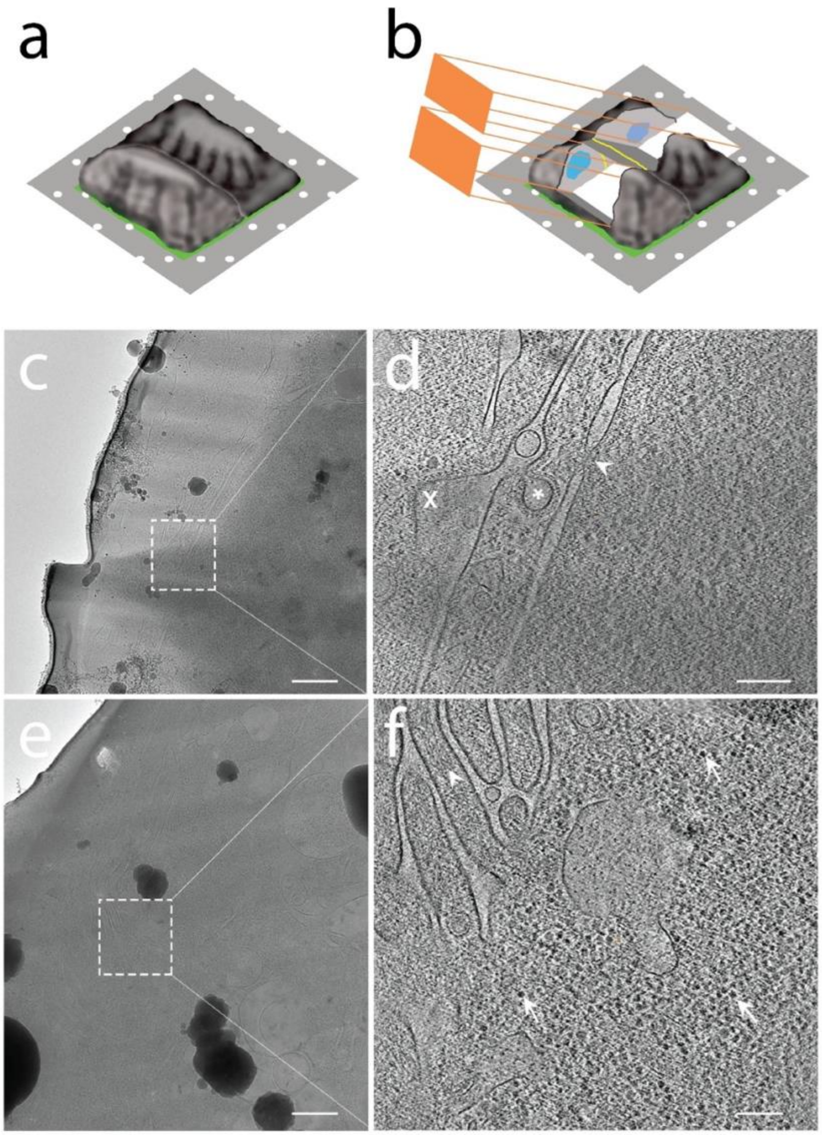

Supplementary Figure S12. In situ cryo-lamellae of cells confined to micropatterns deposited on EM grids. (a) Illustration showing the targeting of a region of interest with tentative cell-cell contacts using the topology of cell doublets confined by a micropattern as it is visible in the cryogenic scanning electron microscope (cryo-SEM) beam of the cryo-FIB dual-beam instrument. (b) Material is removed above and below the target region by milling using the ion beam of the cryo-FIB instrument to generate a lamella thin enough for cryo-ET imaging. The orange rectangles indicate the milling pattern above and below the target cell-cell contact region as identified by cryo-SEM imaging modality. The boundary between the two cells, where the tentative cell-cell contacts are located, is shown as a yellow line. The nuclei are marked in blue. (c) Transmission cryo-EM image of a region adjacent to the area marked in Figure 6a. (d) 2-nm thick virtual slice through a tomogram of the lamella region marked by the white box in c. Two endocytotic events are visible, one in each of the cells. To the right, there is a clathrin-coated pit (marked by an asterisk), to the left, a budding endocytic vesicle (marked by x). Interestingly, the nucleus of the right cell is near the plasma membrane with a nuclear pore clearly visible (marked by an arrowhead). (e) Transmission cryo-EM image of a region from a different lamella prepared from a different PTK1 cell doublet. (f) 2-nm thick virtual slice through a tomogram of the lamella region marked by the white box in E. On the top left, several cellular protrusions, some with clearly visible actin bundles (marked with arrowhead), are present. There is a high density of identifiable ribosomes in this tomogram (some are marked by arrows). Scale bar (c and e) = 500 nm, and (d and f) = 200 nm
